# Nutritional trajectories in gastric cancer patients with early oral feeding

**DOI:** 10.3389/fnut.2025.1656439

**Published:** 2025-10-03

**Authors:** Nan Yao, Haixia Chen, Leyao Han, Meishan Zhang, Meng Yang, Haijun Zhang, Xinglei Wang, Xinman Dou

**Affiliations:** ^1^School of Nursing, Lanzhou University, Lanzhou, Gansu, China; ^2^Department of Nursing, The Second Hospital of Lanzhou University, Lanzhou, Gansu, China; ^3^School of Medicine, Tongji University, Shanghai, China

**Keywords:** gastric cancer, group-based trajectory modeling, nutritional status, nutrition assessment, postoperative period, retrospective studies

## Abstract

**Background/Objectives:**

Evidence on postoperative nutritional dynamics in Chinese gastric cancer (GC) patients is currently limited. This study employs Group-Based Trajectory Modeling (GBTM) to identify Prognostic Nutritional Index (PNI) trajectory patterns and their factors among GC patients under early oral feeding (EOF) management.

**Methods:**

This retrospective study analyzed 124 GC patients undergoing total gastrectomy (2019–2024). PNI trajectories were identified using GBTM, and their associated factors were analyzed via multinomial logistic regression.

**Results:**

Three distinct trajectories emerged: “High nutritional status” (41.9%), “Rapidly declining” (7.3%), and “Decline-Recovery” (50.8%). Compared with the high nutritional status (49.99 ± 4.50), the baseline PNI of the decline-recovery group was lower (44.34 ± 3.57). High Morse Fall Scale (MFS) score (*β* = 0.092, *p* = 0.010), low activities of daily living (ADL) (*β* = −0.655, *p* = 0.009), AJCC Cancer Stage (*β* = 2.238, *p* = 0.002) and vascular and nerve invasion (*β* = 3.540, *p* < 0.001) influence unfavorable trajectories.

**Conclusion:**

Postoperative nutritional trajectories in GC patients managed with EOF are different. Functional impairment (e.g., low ADL, high MFS) and advanced pathological conditions were key determinants of unfavorable nutritional trajectories highlighting the need for targeted monitoring and individualized nutritional interventions for high-risk sub-groups.

## 1 Introduction

GC, a leading global malignancy with high mortality, places a significant burden on public health, particularly in China where substantial fatalities occur annually (1, 2). Surgical resection, including total or subtotal gastrectomy, remains the principal treatment (3, 4); however, it often leads to anatomical and functional gastrointestinal alterations that precipitate malnutrition (5, 6). EOF, initiating oral intake within 24–48 h postoperation as part of Enhanced Recovery After Surgery (ERAS) protocols (7, 8), is the preferred nutritional support strategy aimed at restoring gut microbiota and enhancing recovery. Despite its central role in ERAS, EOF frequently fails to prevent protein-calorie malnutrition and inadequate nutrient intake in these patients (9–15). Given that severe malnutrition is associated with elevated mortality risk yet remains modifiable (5, 16, 17), perioperative nutritional optimization represents a critical therapeutic target.

However, current research on early post-operative nutritional status in Chinese GC patients remains limited. These limitations are primarily threefold: First, primary outcome measures have mainly included length of hospital stay, complication rates, and feeding intolerance (18, 19), and these outcome measures are not directly modifiable, unlike nutritional status itself. Second, many people rely on relevant biomarkers, such as body mass index (BMI) or serum albumin, while ignoring comprehensive assessment tools like PNI that combine serum albumin and lymphocyte count (20–22). Third, at present, most studies on the nutritional status after gastrectomy are cross-sectional studies (15, 23, 24), and it is impossible to dynamically understand the nutritional differences.

To address the first two limitations, we employed the PNI as a comprehensive nutritional indicator. To overcome the third limitation, we utilized longitudinal trajectory analysis. Compared with the traditional cross-sectional study, it can better identify the differences in postoperative nutritional recovery among individuals (25, 26). For example, it can find that some patients recover quickly while others recover slowly. This helps to provide personalized nutritional interventions and care for different patients. Such targeted nutritional optimisation could significantly reduce perioperative morbidity and mortality. Bridging these knowledge gaps would establish clinically implementable strategies to enhance post-gastrectomy prognosis.

## 2 Materials and methods

### 2.1 Study participants

This study was approved by the Second Hospital of Lanzhou University Ethics Committee (Approval No. 2024A-022, 9 January 2025). Inclusion criteria were: (1) age ≥18 years, (2) diagnosed with GC (27, 28) (3) primary laparoscopic total gastrectomy, (4) complete data, (5) nutritional support in the form of EOF. Exclusion criteria were: (1) already in cachexia at diagnosis (29, 30), (2) with distant metastases.

The final analytical comprised 124 adults undergoing total gastrectomy.

### 2.2 Assessment of nutritional status

These items include PNI (PNI = serum albumin (g/L) + 5 × total lymphocyte counts (10^9^/L) (31, 32). This validated composite biomarker evaluates nutritional status, immunological competence, and surgical prognosis (33, 34)), BMI, hemoglobin (g/dL), serum albumin (g/L), leukocyte count (×10^9^/L), lymphocyte count (×10^9^/L), erythrocyte count (×10^12^/L), platelet count (×10^9^/L). The primary outcomes were PNI; secondary outcomes were BMI, serum albumin, hemoglobin, leukocyte, lymphocyte, erythrocyte, and platelet concentrations. Nutritional parameters were assessed at three time points: preoperative baseline (≤48 h pre-surgery), postoperative 7 days (±2 days), postoperative month 1 (±3 days), and postoperative month 3 (±7 days).

### 2.3 Covariates

#### 2.3.1 Sociodemographic variables

The items were composed age (mean ± standard deviation), sex (male, female), education level (illiteracy, primary school, junior/secondary, high school/college), marital status (married/cohabiting, unmarried/separated), occupation (employed, retired, unemployed. Unemployment refers to having no job at present and not receiving a pension), and medical insurance payment method (new rural cooperative medical scheme [NRCMs], urban employee medical insurance, out-of-pocket payment) and residence (urban, rural).

#### 2.3.2 Health condition

Pain intensity was quantified using the verbal numerical rating scale, scored from 0 (no pain) to 10 (maximal pain) (35, 36). ADL was evaluated by the Barthel Index, which evaluated 10 activities: bowel control, bladder management, grooming, feeding, toilet use, transfers, ambulation, dressing, stair climbing, and bathing. Higher ADL scores (maximum 100) indicate greater functional independence (37, 38). Fall risk was measured with the MFS, comprising six parameters: fall history, other diagnoses, ambulatory aid use, intravenous therapy, gait stability, and cognitive status. Elevated MFS scores denote increased fall risk (39, 40). Comorbidities excluded transient conditions (e.g., varicose veins, hemorrhoids) or resolved pathologies (e.g., prior fractures), focusing instead on chronic/systemic diseases: hypertension, chronic hepatitis, diabetes mellitus, extra-gastric malignancies, and HIV/AIDS (41, 42). Pressure injury risk was evaluated using the Braden Scale, where lower scores correlate with higher hospital-acquired pressure injury probability (43, 44).

#### 2.3.3 Health behavior

The items were composed smoking status (current smoker, never smoker), and drinking status (current drinker, never drinker).

#### 2.3.4 Pathological result and surgical conditions

The pathological results include AJCC Cancer Stage (45), degree of differentiation, Lauren classification (46), maximum diameter of the tumor, vascular and nerve invasion. The severity of the surgery was evaluated using the intraoperative blood loss (47).

### 2.4 Statistical analyses

Statistical analyses were performed using R (version 4.3.3). Nutritional trajectory patterns among GC patients adhering to EOF protocols were modelled via GBTM. PNI, the primary normally-distributed continuous outcome, defined the trajectory indicator. Model selection employed Bayesian Information Criterion (BIC) and average posterior probability (AvePP), with lower absolute BIC values indicating superior fit and AvePP > 0.70 confirming adequate classification accuracy. Each trajectory group exceeded 5% compositional representation (26, 48). The final 3-class model was selected based on its optimal statistical fit and clinical interpretability. This model demonstrated a superior balance in both BIC and Akaike Information Criterion (AIC) values (see Supplementary Table S2 for detailed values) and achieved average posterior probabilities (AvePP) all above 0.70, indicating classification is acceptable.

Trajectory classifications derived from the final model constituted the multinomial dependent variable. Determinants were analysed using multinomial logistic regression in SPSS (version 27.0.1).

Sample size justification was established through an *a priori* power analysis using PASS 2021 software, configured to detect anticipated group differences in longitudinal PNI trajectories. The analysis parameters included a target power (1-*β*) of 0.80, *α* level of 0.05, a medium effect size (*f* = 0.25), and an estimated correlation of *ρ* = 0.4 between repeated measurements based on preliminary data and clinical understanding of PNI variability. These inputs indicated a minimum requirement of 72 participants. The final sample included 124 participants, exceeding this threshold. Post-hoc analysis confirmed achieved power of 0.96, indicating robust capability to identify significant trajectory differences among groups.

## 3 Results

### 3.1 Characteristics of participants

All the patients are married. The study comprised 24.2% female. Most patients had favorable functional status; however, 21.8% of patients had comorbidities before surgery (Table 1). The results are detailed in Supplementary Table S1.

**Table 1:** Demographic and clinical characteristics in each nutritional status group.

Characteristics	Class 1 (*n* = 63)	Class 2 (*n* = 9)	Class 3 (*n* = 52)	Total (*n* = 124)	*p*
ADL					
≥80	63 (100.0)	5 (55.6)	52 (100.0)	120 (96.8)	<0.001^b^
<80	0 (0)	4 (44.4)	0 (0)	4 (3.2)	
MFS					
0–20	63 (100.0)	4 (44.4)	52 (100.0)	119 (96.0)	<0.001^b^
21–50	0 (0)	5 (55.6)	0 (0)	5 (4.0)	
Braden					
15–20	7 (11.1)	6 (66.7)	9 (17.3)	14 (11.3)	<0.001^a^
21–35	56 (88.9)	3 (33.3)	43 (82.7)	110 (88.7)	
AJCC cancer stage					
Early	19 (30.1)	1 (11.1)	16 (30.8)	36 (29.0)	<0.001^a^
Middle and late stages	35 (55.6)	1 (11.1)	28 (53.8)	64 (51.6)	
Late stage	9 (14.3)	7 (77.8)	8 (15.4)	24 (19.4)	
Lauren classification					
Intestinal type	19 (30.1)	0 (0)	23 (44.2)	42 (33.9)	<0.001^b^
Hybrid type	10 (15.9)	6 (66.7)	4 (7.7)	20 (16.1)	
Diffuse type	34 (54.0)	3 (33.3)	25 (48.1)	62 (50.0)	
Vascular and nerve invasion					
No	41 (65.1)	2 (22.2)	47 (90.4)	90 (72.6)	<0.001^a^
Yes	22 (34.9)	7 (77.8)	5 (9.6)	34 (27.4)	

Class 1: “Decline-Recovery (V-shaped)”; Class 2: “Rapidly declining”; Class 3: “High nutritional status”.

a Chi-square test.

b Corrected chi-square test.

### 3.2 Nutritional status at each time point in each group

Table 2 shows the changes of various nutritional indicators in these three groups. The complete table can be found in Supplementary Table S2. Compared with the class 3, the class 2 and 1 had lower PNI (T0: *p* < 0.001; T1: *p* < 0.001; T2: *p* < 0.001; T3: *p* < 0.001), serum albumin (T0: *p* < 0.001; T1: *p* < 0.001; T2: *p* < 0.001; T3: *p* < 0.001), lymphocyte Counts (T0: *p* = 0.004; T1: *p* < 0.001; T2: *p* = 0.002; T3: *p* = 0.001) and Erythrocyte counts (T0: *p* = 0.032; T1: *p* = 0.001; T2: *p* = 0.002; T3: *p* = 0.01).

**Table 2:** Nutritional status at each time point in each nutritional status group.

Time	Variables	*p*
Before surgery(T0)	PNI	<0.001
	Lymphocyte Count	0.004
	Red Blood Cell Count	0.032
	Albumin	<0.001
1 week after the operation (T1)	PNI	<0.001
	Lymphocyte Count	<0.001
	Red Blood Cell Count	0.001
	Hemoglobin	0.008
	Albumin	<0.001
1 month after surgery (T2)	PNI	<0.001
	Lymphocyte Count	0.002
	Red Blood Cell Count	0.002
	Hemoglobin	<0.001
	Albumin	<0.001
3 months after surgery (T3)	PNI	<0.001
	Lymphocyte Count	0.001
	Red Blood Cell Count	0.01
	Albumin	<0.001

*p* value was examined by Kruskal-Wallis test to compare differences among the three groups.

### 3.3 The changes in nutritional status at different trajectories

**Figure 1:**
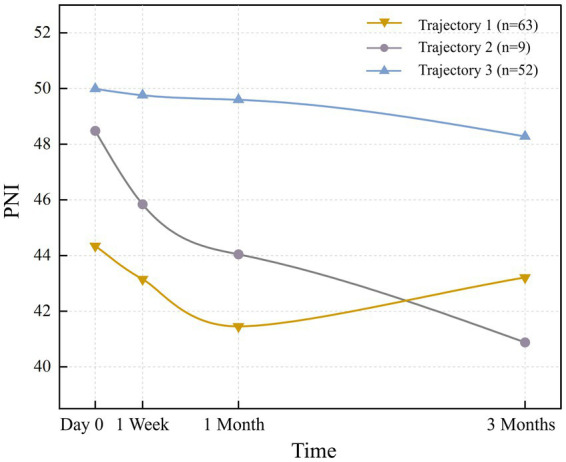
Nutritional status trajectories.

The 3-class solution, retained for its optimal clinical interpretability and statistical robustness (Supplementary Table S3), consisted of the following trajectories: ‘High nutritional status’ (Class 3), ‘Rapidly declining’ (Class 2), and ‘Decline-Recovery (V-shaped)’ (Class 1). There were 52 patients (41.9%) with an average PNI of 49.9. Trajectory 2 (‘Rapidly declining’) comprised 9 patients (7.3%) with a steep decline in PNI (mean baseline: 44.34 ± 3.57). The 63 patients (50.8%) in trajectory 1 “Decline-Recovery (V-shaped)” had the smallest PNI at day 0 (preoperative), and declined at a slower rate than trajectory 2, reaching a bottom between 1 month after the operation, but then it increased gradually and exceeds trajectory 2. Additionally, all three trajectories exhibited a downward trend within 1 month after the operation, which reversed or stabilized thereafter, resulting in divergent patterns (downward vs. upward). The nutritional status trajectories for each trajectory are shown in Figure 1.

### 3.4 Factors related to nutritional status trajectories

Multiple logistic regression was used to identify significant factors affecting nutritional status trajectories (Table 3). Detailed data can be found in Supplementary Table S4. The results showed that ADL, MFS, AJCC Cancer Stage and vascular and nerve invasion were influential factors in the postoperative nutritional status trajectory of GC patients.

**Table 3:** Significant factors related to nutritional status trajectories.

Variable	*p*	OR (95% CI)
ADL	0.009	0.519 (0.318, 0.850)
MFS	0.010	1.096 (1.022, 1.176)
AJCC Cancer Stage	0.002	9.377 (2.207, 39.835)
Vascular and nerve invasion	<0.001	34.479 (5.479, 216.693)

### 3.5 Pairwise comparison of survival rates between trajectories

The Kaplan–Meier survival curves of the three trajectories are shown in Supplementary Figure S1, and the median overall survival for each group is summarized in Supplementary Table S5. The Log-rank test showed that there were significant differences in the survival distribution between trajectories (*p* < 0.001). The paired comparison results further indicated that the prognosis of patients in class 2 was significantly worse than that in class 1 (χ^2^ = 26.171, *p* < 0.001) and class 3 (χ^2^ = 35.581, *p* < 0.001). However, there was no statistically significant difference in survival rate between Class 1 and Class 3 (χ^2^ = 2.356, *p* = 0.125). The median survival time of Trajectory 2 was 281 days. As more than 50% of the individuals in Trajectory 1 and Trajectory 3 had not experienced any events by the end of the study, the median survival time could not be calculated (not reached).

## 4 Discussion

This study provides the first evidence of differences in nutritional trajectories among Chinese GC patients under EOF management. These findings establish a foundation for stratifying malnutrition-risk subpopulations. In this study, while no significant intergroup differences in BMI were observed, PNI values differed significantly (*p* < 0.001). This indicates that compared with BMI, PNI has higher sensitivity in detecting the trajectory of nutritional status. Overall, this study has the following findings:

A significant proportion (50.8%; Trajectory 1) exhibited a distinct V-shaped nutritional trajectory. One month after the operation, the nutritional status decreased to the lowest point (PNI = 41.44 ± 3.93). This decline is attributable to three primary factors: (1) Impaired gastric secretion and reduced surface area compromise nutrient absorption, leading to deficiencies (49, 50). (2) Total gastrectomy leads to deficient ghrelin secretion—primarily due to the removal of ghrelin-producing fundic glands and the disruption of neuroregulation caused by vagus nerve transection—which contributes significantly to reduced appetite after surgery (51–53). (3) The Surgical Stress Response leads to a significant loss of protein and abnormal metabolism of nutrients (54). Although nutritional status often begins to improve approximately 1 month postoperatively, this recovery is largely attributed to behavioral adaptations such as frequent small meals, which help compensate for reduced gastric capacity, rather than a restoration of ghrelin levels.

In addition, 41.9% (Trajectory 3) of the patients maintained a high nutritional status with PNI > 49. Although the proportion of this subgroup is lower compared to Trajectory 1, its clinical significance cannot be ignored. This is because a subset of patients maintains this favorable trajectory regardless of tumor progression status (55, 56). In the High nutritional status, patients sustained optimal postoperative nutritional status. This group exhibited maximal ADL scores. These observations suggest that higher activities of daily living may represent protective factors. For Trajectory 2 with the lowest proportion (7.3%), patients showed a rapid downward trend in nutritional indicators, which was consistent with other studies (55, 56).

The three PNI trajectories identified in this study have significant clinical translational value. This model can prospectively identify 7.3% of high-risk patients with “rapid decline type,” thus enabling early intervention to curb their nutritional deterioration and improve their poor prognosis. Meanwhile, different trajectories provide a basis for individualized management: for patients with the “down-and-recovery type,” it is necessary to focus on protecting them through the one-month postoperative nutritional trough, while for patients with the “high nutritional status type,” the standard procedures can be safely followed. In addition, this stratification tool helps optimize the allocation of medical resources, prioritizing energy and resources on the most high-risk groups, and ultimately achieving a transformation from standardized care to precise nutrition management.

This study employed multiple logistic regression analysis to identify key determinants of nutritional trajectories. AJCC Cancer Stage and vascular and nerve invasion types significantly affect the development trajectory (*p* < 0.01), which is consistent with the conclusions of previous studies (17, 57, 58). However, the critical implication of our findings lies not merely in identifying this high-risk population, but in interrogating what modifiable factors might mitigate their risk or, conversely, what reversible deficits propel them toward the worst outcomes. Our analysis reveals that beyond fixed tumor characteristics, functional and physiological markers—specifically, low ADL scores and high MFS—are powerfully associated with poor nutritional trajectories. This is a pivotal finding because unlike tumor stage, functional capacity and frailty are dynamic and potentially improvable through targeted prehabilitation and rehabilitation programs.

Therefore, our findings on the association between functional impairment (low ADL, high MFS) and unfavorable nutritional trajectories, being observational, highlight potential targets for intervention but cannot prove efficacy. Theoretically, strategies aimed at improving functional status—such as multi-component exercise programs to enhance muscle strength and ADL (59–62). Vestibular rehabilitation therapy to potentially reduce fall risk (63, 64). These hypotheses, however, must be rigorously tested in prospective, interventional studies to determine if modifying these risk factors can indeed causally improve nutritional outcomes in high-risk patients.

A deteriorated low PNI status indicates a poor prognosis for patients, which is consistent with the results of other studies (65–68). This study identified a high-risk population with significant clinical significance (Trajectory 2, *p* < 0.001), whose survival rate was lower (median survival time: 281 days < 1 year), which was consistent with the results of other studies (4), but more importantly, it identifies this high-risk population prospectively through its dynamic trajectory pattern. This finding suggests that clinical intervention should focus on this group of patients, improving their poor prognosis through enhanced nutritional support and close monitoring. It is worth noting that although the evolution processes of trajectories 1 and 3 are different, there is no difference in long-term survival rates (χ^2^ = 2.356, *p* = 0.125), suggesting that the adverse trends that emerged in the early stage are not irreversible. If timely intervention is carried out within the critical time window after surgery to reverse the downward trend, the prognosis of the patient can still reach a level comparable to that of the initially stable patient. Therefore, implementing precise intervention for high-risk groups can optimize the allocation of clinical resources and improve the overall survival outcomes of patients.

This study highly supports the core recommendations of the ESPEN surgical nutrition guidelines and provides empirical supplements for Chinese patients with GC (24). The guideline strongly recommends EOF. This study confirmed that even with the implementation of EOF, patients still presented three different nutritional trajectories, suggesting significant heterogeneity in nutritional recovery and emphasizing the need for dynamic monitoring and individualized intervention. Consistent with the results of multi-center studies such as Deftereos (69), this study found that even under the standardized implementation of EOF, there was still significant heterogeneity in nutritional trajectories. Matsunaga et al. (70) emphasized that among elderly patients with gastric cancer, the Geriatric Nutritional Risk Index has the highest prognostic predictive value among multiple inflammatory and nutritional indicators. Although GNRI was not directly adopted in this study, it was still found that PNI has a high sensitivity in identifying high-risk populations and predicting survival outcomes.

This research provides a crucial methodological advancement. The GBTM was applied to quantify the nutritional trajectory of Chinese GC patients. This method overcomes the inherent limitations of the cross-sectional study design. Our GBTM analysis revealed significant nutritional trajectory differences, which the PNI effectively classified into distinct subgroups. The identified trajectory patterns provide critical insights into nutritional progression dynamics. These findings enable the formulation of precision interventions and targeted support strategies to optimise patient nutritional outcomes.

However, this study has several limitations. First, its retrospective, single-center design may limit the generalizability of the findings. This design also restricted our data collection to what was routinely available in medical records, which precluded the assessment of potentially significant unmeasured confounders—such as socioeconomic status, dietary adherence, and family support—that could affect outcomes (71, 72). This absence of more nuanced data prevents a more granular analysis of the mechanisms behind the observed trajectories. Secondly, the lack of quantitative dietary intake records, including actual oral intake, actual intake of vitamins and minerals, and supplement usage, hinders the mechanism explanation of the observed trajectory differences. Third, this study is limited by its short-term focus (up to 3 months post-operation), which fails to capture longer-term nutritional changes. High rates of loss to follow-up beyond this point, together with strong confounders such as chemotherapy and tumor progression, make it difficult to draw reliable conclusions from retrospective data. Future prospective studies should implement systematic follow-ups and detailed records of dietary intake, body composition, and treatment to better map nutritional recovery. Finally, including only patients with complete follow-up data may introduce selection bias, as those with missing data might have worse conditions or poorer compliance, potentially limiting the extrapolation of our results. Therefore, future multicenter prospective studies with frequent monitoring and detailed dietary assessment are needed to validate and extend our findings.

## 5 Conclusion

The development trajectory of nutritional status in patients with EOF after GC surgery is different. These differences help identify high-risk patients, and healthcare providers can offer precisely customized nutritional intervention measures.

## Data Availability

The raw data supporting the conclusions of this article will be made available by the authors, without undue reservation.
