# Supplementary material for: Nutritional trajectories in gastric cancer patients with early oral feeding
Source: Front Nutr. 2025 Oct 3;12:1656439. doi: 10.3389/fnut.2025.1656439 (PMC12531070; doi:10.3389/fnut.2025.1656439)
Supplement: Supplementary file 1 [file Table_1.DOCX]

Supplementary Material

# Table S1. Demographic and clinical characteristics in each nutritional status group.

| Characteristics | Class 1(n=63) | Class 2(n=9) | Class 3(n=52) | Total(n=124) | Statistic | *p* |
| --- | --- | --- | --- | --- | --- | --- |
| ADL |  |  |  |  | 132.984 | **＜0.001^b^** |
| ≥80 | 63 (100.0) | 5 (55.6) | 52 (100.0) | 120 (96.8) |  |  |
| ＜80 | 0 (0) | 4 (44.4) | 0 (0) | 4 (3.2) |  |  |
| MFS |  |  |  |  | 156.251 | **＜0.001^b^** |
| 0-20 | 63 (100.0) | 4 (44.4) | 52 (100.0) | 119 (96.0) |  |  |
| 21-50 | 0 (0) | 5 (55.6) | 0 (0) | 5 (4.0) |  |  |
| Braden |  |  |  |  | 51.29 | **＜0.001^a^** |
| 15-20 | 7 (11.1) | 6 (66.7) | 9 (17.3) | 22 (17.7) |  |  |
| 21-35 | 56 (88.9) | 3 (33.3) | 43 (82.7) | 102 (82.3) |  |  |
| Pain |  |  |  |  | 0.179 | 0.914^b^ |
| No pain | 62 (98.4) | 9 (100.0) | 51 (98.1) | 122 (98.4) |  |  |
| Pain | 1 (1.6) | 0 (0) | 1 (1.9) | 2 (1.6) |  |  |
| Age |  |  |  |  | 96.028 | 0.174^a^ |
| 18-50 | 19 (30.2) | 5 (55.6) | 18 (34.6) | 42 (33.9) |  |  |
| 51-79 | 44 (69.8) | 4 (44.4) | 34 (65.4) | 82 (66.1) |  |  |
| Sex, n (%) |  |  |  |  | 0.549 | 0.760^a^ |
| Female | 17 (27.0) | 2 (22.2) | 11 (21.2) | 30 (24.2) |  |  |
| Male | 46 (73.0) | 7 (77.8) | 41 (78.8) | 94 (75.8) |  |  |
| Edu |  |  |  |  | 8.156 | 0.227^a^ |
| Uneducated | 11 (17.5) | 3 (33.4) | 8 (15.4) | 22(17.7) |  |  |
| Primary | 35 (55.6) | 4 (44.4) | 19 (36.5) | 58(46.8) |  |  |
| Junior/Secondary | 11 (17.5) | 1 (11.1) | 18 (34.6) | 30(24.2) |  |  |
| High school/college | 6 (9.4) | 1 (11.1) | 7 (13.5) | 14(11.3) |  |  |
| Marital status, n (%) |  |  |  |  | - | - |
| Married | 63 (100.0) | 9 (100.0) | 52 (100.0) | 124 (100.0) |  |  |
| Residence, n (%) |  |  |  |  | 0.863 | 0.649^a^ |
| Urban | 14 (22.2) | 1 (11.1) | 9 (17.3) | 24 (19.4) |  |  |
| Rural | 49 (77.8) | 8 (88.9) | 43 (82.7) | 100 (80.6) |  |  |
| Work |  |  |  |  | 2.542 | 0.637^b^ |
| Employed | 56 (88.9) | 8 (88.9) | 41 (78.8) | 105 (84.7) |  |  |
| Unemployed | 1 (1.6) | 0 (0) | 1 (2.0) | 2 (1.6) |  |  |
| Retired | 6 (9.5) | 1 (11.1) | 10 (19.2） | 17 (13.7) |  |  |
| Insurance |  |  |  |  | 0.813 | 0.937^b^ |
| Medical insurance | 17 (27.0) | 2 (22.2) | 16 (30.8) | 35 (28.2) |  |  |
| New rural cooperative | 44 (69.8) | 7 (77.8) | 35 (67.3) | 86 (69.4) |  |  |
| Out-of-pocket expenses | 2 (3.2) | 0 (0) | 1 (1.9) | 3 (2.4) |  |  |
| Smoking, n (%) |  |  |  |  | 0.766 | 0.682^a^ |
| Non-smoker | 50 (79.4) | 6 (66.7) | 41 (78.8) | 97 (78.2) |  |  |
| Smoker | 13 (20.6) | 3 (33.3) | 11 (21.2) | 27 (21.8) |  |  |
| Alcohol intake, n (%) |  |  |  |  | 0.383 | 0.826^a^ |
| Non-drinker | 54 (85.7) | 7 (77.8) | 44 (84.6) | 105 (84.7) |  |  |
| Drinker | 9 (14.3) | 2 (22.2) | 8 (15.4) | 19 (15.3) |  |  |
| Comorbidity, n (%) |  |  |  |  | 0.101 | 0.951^a^ |
| No | 50 (79.4) | 7 (77.8) | 40 (76.9) | 97 (78.2) |  |  |
| Yes | 13 (20.6) | 2 (22.2) | 12 (23.1) | 27 (21.8) |  |  |
| AJCC Cancer Stage |  |  |  |  | 21.309 | **＜0.001^a^** |
| Early | 19 (30.1) | 1 (11.1) | 16 (30.8) | 36 (29.0) |  |  |
| Middle and late stages | 35 (55.6) | 1 (11.1) | 28 (53.8) | 64 (51.6) |  |  |
| Late stage | 9 (14.3) | 7 (77.8) | 8 (15.4) | 24 (19.4) |  |  |
| Degree of differentiation |  |  |  |  | 9.275 | 0.320^b^ |
| Low differentiation | 29 (46.0) | 5 (55.6) | 21 (40.4) | 55 (44.3) |  |  |
| Moderately low differentiation | 16 (25.4) | 2 (22.2) | 21 (40.4) | 39 (31.5) |  |  |
| Moderate differentiation | 10 (15.9) | 2 (22.2) | 5 (9.6) | 17 (13.7) |  |  |
| Moderately high differentiation | 8 (12.7) | 0 (0) | 3 (5.8) | 11 (8.9) |  |  |
| Highly differentiated | 0 (0) | 0 (0) | 2 (3.8) | 2 (1.6) |  |  |
| Lauren classification |  |  |  |  | 22.238 | **＜0.001^b^** |
| Intestinal type | 19 (30.1) | 0 (0) | 23 (44.2) | 42 (33.9) |  |  |
| Hybrid type | 10 (15.9) | 6 (66.7) | 4 (7.7) | 20 (16.1) |  |  |
| Diffuse type | 34 (54.0) | 3 (33.3) | 25 (48.1) | 62 (50.0) |  |  |
| Maximum diameter of the tumor |  |  |  |  | 51.628 | 0.200^a^ |
| 1-5 | 53 (84.1) | 7 (77.8) | 39 (75.0) | 99 (79.8) |  |  |
| 6-13 | 10 (15.9) | 2 (22.2) | 13 (25.0) | 25 (20.2) |  |  |
| Vascular and nerve invasion |  |  |  |  | 21.532 | **＜0.001^a^** |
| No | 41 (65.1) | 2 (22.2) | 47 (90.4) | 90 (72.6) |  |  |
| Yes | 22 (34.9) | 7 (77.8) | 5 (9.6) | 34 (27.4) |  |  |
| Intraoperative blood loss |  |  |  |  | 32.858 | 0.107^a^ |
| 0-1000 | 62 (98.0) | 8 (88.9) | 51 (98.0) | 121 (97.6) |  |  |
| 1000-1500 | 1 (2.0) | 1 (11.1) | 1 (2.0) | 3 (2.4) |  |  |

Class 1: “Decline-Recovery (V-shaped)”; Class 2: “Rapidly declining”; Class 3: “High nutritional status”.

^a^ chi-square test.

^b^ Corrected chi-square test.

| Variables | Overall (N=124) | Class 1(n=63) | Class 2(n=9) | Class 3(n=52) | z | *p* |
| --- | --- | --- | --- | --- | --- | --- |
| Before surgery(T0) |  |  |  |  |  |  |
| BMI | 22.12 ± 3.36 | 22.09 ± 2.83 | 21.36 ± 2.74 | 22.3 ± 4.02 | 2.661 | 0.264 |
| PNI | 47.01 ± 6.03 | 44.34 ± 3.57 | 48.48 ± 14.7 | 49.99 ± 4.50 | 35.362 | ＜0.001 |
| Leukocyte Count | 6.10 ± 2.34 | 6.30 ± 2.80 | 6.80 ± 2.11 | 5.74 ± 1.65 | 1.402 | 0.496 |
| Lymphocyte Count | 1.44 ± 0.57 | 1.27 ± 0.46 | 1.58 ± 1.01 | 1.62 ± 0.53 | 11.217 | 0.004 |
| Red Blood Cell Count | 4.36 ± 0.74 | 4.19 ± 0.72 | 4.33 ± 1.04 | 4.57 ± 0.67 | 6.899 | 0.032 |
| Hemoglobin | 130.54 ± 27.25 | 125.52 ± 28.61 | 126 ± 32.88 | 137.4 ± 23.32 | 4.339 | 0.114 |
| Platelets | 217.29 ± 106.43 | 215.57 ± 115.89 | 183.56 ± 93.11 | 225.21 ± 96.76 | 2.071 | 0.355 |
| Albumin | 39.8 ± 5.23 | 37.98 ± 3.59 | 40.58 ± 12.79 | 41.87 ± 4.00 | 24.835 | ＜0.001 |
| One week after the operation (T1) |  |  |  |  |  |  |
| BMI | 21.26 ± 3.28 | 21.26 ± 2.85 | 20.80 ± 2.67 | 21.78 ± 3.85 | 1.673 | 0.433 |
| PNI | 46.09 ± 5.47 | 43.15 ± 2.74 | 45.86 ± 12.99 | 49.76 ± 2.98 | 70.982 | ＜0.001 |
| Leukocyte Count | 6.00 ± 1.81 | 6.06 ± 2.00 | 5.61 ± 1.45 | 5.98 ± 1.65 | 0.01 | 0.995 |
| Lymphocyte Count | 1.35 ± 0.47 | 1.18 ± 0.35 | 1.48 ± 0.67 | 1.50 ± 0.5 | 15.415 | ＜0.001 |
| Red Blood Cell Count | 4.18 ± 0.63 | 4.02 ± 0.57 | 4.14 ± 0.96 | 4.01 ± 0.57 | 13.652 | 0.001 |
| Hemoglobin | 124.06 ± 21.93 | 118.74 ± 21.91 | 119.58 ± 28.16 | 130.82 ± 18.81 | 9.774 | 0.008 |
| Platelets | 216/98 ± 134.8 | 208.80 ± 140.88 | 182.36 ± 118.55 | 231.34 ± 128.96 | 2.496 | 0.287 |
| Albumin | 39.32 ± 4.78 | 36.92 ± 2.71 | 38.60 ± 12.61 | 42.16 ± 2.49 | 58.377 | ＜0.001 |
| 1 month after surgery (T2) |  |  |  |  |  |  |
| BMI | 20.69 ± 3.59 | 20.47 ± 3.57 | 20.56 ± 2.66 | 20.98 ± 3.79 | 0.966 | 0.617 |
| PNI | 45.05 ± 7.28 | 41.44 ± 3.93 | 44.04 ± 19.19 | 49.59 ± 3.73 | 62.416 | ＜0.001 |
| Leukocyte Count | 5.96 ± 2.81 | 5.88 ± 3.07 | 5.00 ± 1.72 | 6.21 ± 2.62 | 2.467 | 0.291 |
| Lymphocyte Count | 1.28 ± 0.54 | 1.12 ± 0.45 | 1.34 ± 0.44 | 1.45 ± 0.61 | 12.286 | 0.002 |
| Red Blood Cell Count | 4.00 ± 0.71 | 3.81 ± 0.63 | 3.88 ± 1.14 | 4.25 ± 0.64 | 12.184 | 0.002 |
| Hemoglobin | 118.32 ± 21.61 | 112.48 ± 20.01 | 112.33 ± 32.87 | 126.44 ± 18.82 | 13.872 | ＜0.001 |
| Platelets | 216.81 ± 99.86 | 205.62 ± 100.88 | 181 ± 68.82 | 236.56 ± 100.77 | 4.124 | 0.127 |
| Albumin | 38.65 ± 6.64 | 35.81 ± 3.66 | 37.34 ± 18.8 | 42.33 ± 3.26 | 53.317 | ＜0.001 |
| 3 months after surgery (T3) |  |  |  |  |  |  |
| BMI | 20.00 ± 3.68 | 19.93 ± 3.74 | 19.82 ± 3.20 | 20.12 ± 3.76 | 0.451 | 0.798 |
| PNI | 45.13 ± 6.60 | 43.13 ± 4.07 | 40.88 ± 17.48 | 48.28 ± 4.34 | 31.621 | ＜0.001 |
| Leukocyte Count | 4.78 ± 2.45 | 4.73 ± 2.95 | 4.51 ± 1.58 | 4.89 ± 1.88 | 1.948 | 0.378 |
| Lymphocyte Count | 1.29 ± 0.50 | 1.14 ± 0.45 | 1.18 ± 0.57 | 1.48 ± 0.50 | 13.502 | 0.001 |
| Red Blood Cell Count | 3.83 ± 0.71 | 3.72 ± 0.63 | 3.52 ± 0.93 | 4.02 ± 0.72 | 9.194 | 0.01 |
| Hemoglobin | 116.83 ± 19.52 | 114.56 ± 16.51 | 109.44 ± 26.84 | 120.87 ± 21.02 | 4.284 | 0.117 |
| Platelets | 168.82 ± 80.78 | 167.71 ± 81.48 | 121.78 ± 55.64 | 178.29 ± 81.86 | 4.813 | 0.09 |
| Albumin | 38.99 ± 4.90 | 37.44 ± 3.8 | 39.01 ± 11.91 | 40.87 ± 3.40 | 22.363 | ＜0.001 |

# Table S2. Nutritional status at each time point in each nutritional status group.

z statistic from Kruskal-Wallis test.

p value was examined by Kruskal-Wallis test to compare differences among the three groups.

Bold prompt p < 0.05.

# Table S3. Fit statistics of trajectory analysis

| **Fit statistics** | **Number of class** | | | | |
| --- | --- | --- | --- | --- | --- |
|  | **1** | **2** | **3** | **4** | **5** |
| **BIC** | 3261.949 | 3226.545 | 3092.266 | 3128.371 | 2847.157 |
| **AIC** | 3249.330 | 3192.892 | 3037.580 | 3044.240 | 2741.993 |
| **CAIC** | 3264.949 | 3234.545 | 3105.266 | 3148.371 | 2872.157 |
| **SSBIC** | 3252.427 | 3201.152 | 3051.003 | 3064.891 | 2767.806 |
| **HQIC** | 3254.283 | 3206.102 | 3059.046 | 3077.264 | 2783.273 |
| **Average posterior probabilities (AVePP)** |  | | | | |
| Class 1 | 1 | 0.88 | 0.92 | 0.91 | 0.87 |
| Class 2 |  | 0.97 | 0.93 | 0.81 | 0.91 |
| Class 3 |  |  | 0.90 | 0.89 | 0.92 |
| Class 4 |  |  |  | 0.93 | 0.96 |
| Class 5 |  |  |  |  | 1 |

Abbreviation: BIC: Bayesian information criterion, AIC: Akaike’s information criterion. The 5-class model was rejected due to clinically implausible group segmentation.

# Table S4. Significant factors related to nutritional status trajectories.

| **Variable** | ***β*** | ***Std. Err.*** | ***Wald chi-square*** | ***p*** | **OR (95 % CI)** |
| --- | --- | --- | --- | --- | --- |
| ADL | -0.655 | 0.251 | 6.806 | **0.009** | 0.519 (0.318, 0.850) |
| MFS | 0.092 | 0.036 | 6.659 | **0.010** | 1.096 (1.022, 1.176) |
| Braden | -0.247 | 0.160 | 2.389 | 0.122 | 0.781 (0.571, 1.069) |
| pain | -0.607 | 0.956 | 0.403 | 0.525 | 0.545, (0.084, 3.547) |
| Sex | 0.063 | 0.598 | 0.011 | 0.917 | 1.065 (0.330, 3.435) |
| Age | 0.019 | 0.025 | 0.578 | 0.447 | 1.019 (0.971, 1.069) |
| Edu | -0.361 | 0.306 | 1.394 | 0.238 | 0.697 (0.383, 1.269) |
| Residence | 1.186 | 0.815 | 2.116 | 0.146 | 3.273 (0.662, 16.175) |
| Occupation | -0.725 | 0.480 | 2.279 | 0.131 | 0.484 (0.189, 1.242) |
| Insurance | 0.057 | 0.575 | 0.010 | 0.921 | 1.058 (343, 3.268) |
| Smoking | 0.105 | 0.681 | 0.024 | 0.877 | 1.111 (0.292, 4.220) |
| Alcohol intake | -0.501 | 0.774 | 0.419 | 0.517 | 0.606 (0.133, 2.764) |
| Comorbidity | 0.379 | 0.578 | 0.431 | 0.512 | 1.461 (0.471, 4.534) |
| AJCC Cancer Stage | 2.238 | 0.738 | 9.197 | **0.002** | 9.377 (2.207, 39.835) |
| Degree of differentiation | -0.424 | 0.521 | 0.662 | 0.416 | 0.655 (0.236, 1.817) |
| Lauren classification | 0.336 | 0.500 | 0.451 | 0.502 | 1.399 (0.525, 3.731) |
| Maximum diameter of the tumor | 0.149 | 0.152 | 0.966 | 0.326 | 1.161 (0.862, 1.565) |
| Vascular and nerve invasion | 3.540 | 0.938 | 14.251 | **＜0.001** | 34.479 (5.479, 216.693) |
| Intraoperative blood loss | 0.002 | 0.001 | 3.488 | 0.062 | 1.002 (1.000, 1.004) |

Abbreviations: Std. Err., standard error.


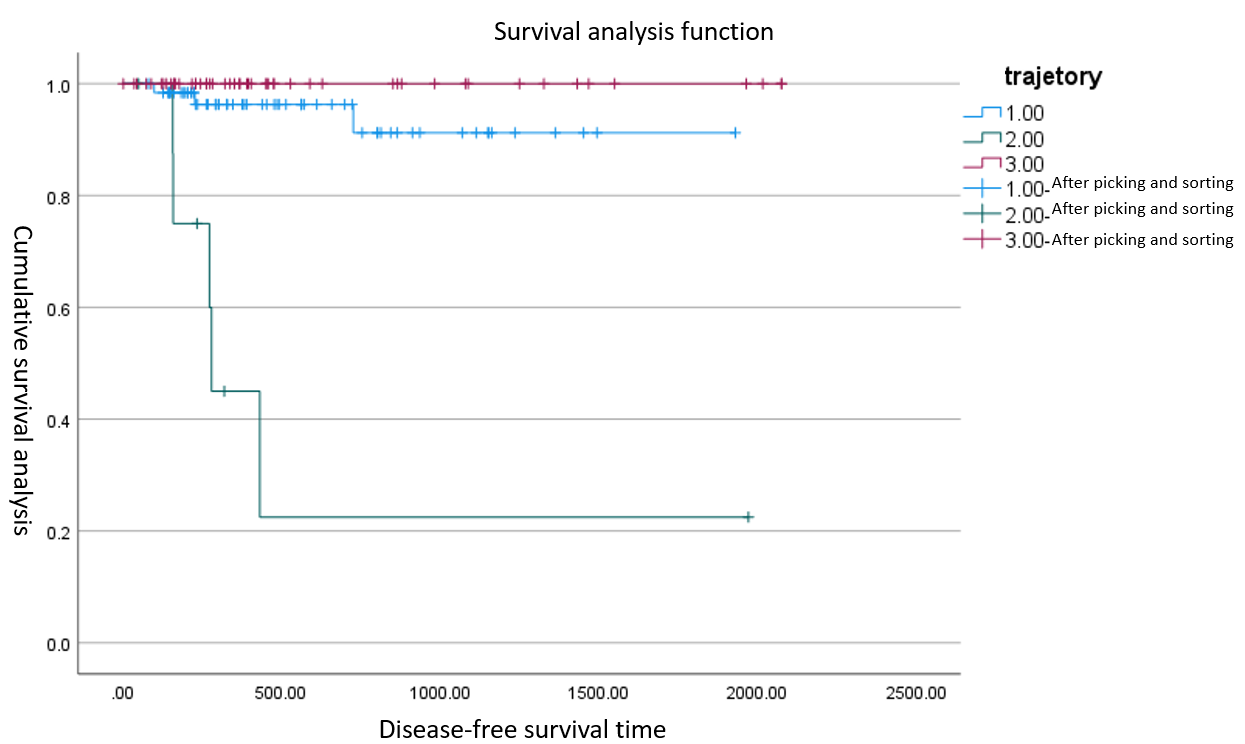


# Figure S1. Kaplan-Meier survival curves for the three identified tragectories.

# Table S5. Pairwise comparisons of survival among trajectory groups using the Log-rank test.

| Comparison | Chi-Square (χ²) | *p* |
| --- | --- | --- |
| Class 1 vs. Class 2 | 26.171 | **< 0.001** |
| Class 1 vs. Class 3 | 2.356 | 0.125 |
| Class 2 vs. Class 3 | 35.581 | **< 0.001** |
